# News

**DOI:** 10.1155/S1463924600000122

**Published:** 2000


Journal of Automated Methods & Management in Chemistry, Vol. 22, No. 3 (May–June 2000) p. 96

### News

### Productivity awards for drug safety and efficacy testing announced

*Hopkinton, MA, December 1999:* The 1999 Laboratory Productivity Awards were presented by Zymark Corporation to 93 individuals at this year's International Symposium of Laboratory Automation and Robotics (ISLAR). These awards are designed to acknowledge the tremendous levels of productivity attained through automation in Pharmaceutical Quality Assurance, Quality Control and R&D laboratories. This year's awards are representative of the more than 8 million tests performed to ensure the dissolution testing and potency of the medicines produced today. Individuals honoured have chosen automation as a way to meet the productivity challenges of their organizations while reducing retesting and overall costs.

Increasing productivity while adhering to the stringent guidelines and deadlines that are instituted are the main challenges of Quality Assurance, Quality Control and Research and Development laboratories throughout the pharmaceutical industry. Constant struggles to find ways to perform more efficiently and more economically have left these laboratories in search of support. It is with these challenges in mind that Zymark Corporation has developed laboratory automation products, services and programs to help the laboratories meet the challenges of today and into the future.

Initiated in 1996, The Zymark Productivity Club supports those who champion the technology of laboratory automation and have proven that automation is essential for the success of their work. After joining, members are invited to attend technology meetings to discuss new developments and provide insight into future challenges for pharmaceutical dosage from automation.

'We believe that a partnership between Zymark and Productivity Club members increases the knowledge of everyone', explains Guy Dechamps, Zymark's Vice President of Worldwide Sales. 'Together, we gain valuable insight and are able to work to meet the challenges of the emerging technologies that will impact the pharmaceutical industry'.

The Zymark Productivity Program Awards consist of four achievement levels based on the throughput of the laboratory workstation or system and based on the diversity of the products being run. These levels are: Acceptance (validated and on line); Priority (1000–10 000 tablet/capsules); Gold (10 000–100 000 tablets/capsules); Platinum (100 000–1 million tablets/capsules); and Hall of Fame (1 million tablets/capsules). This year, for the first time, eight laboratories have reached the Platinum level. They include laboratories at Bristol Myers Squibb, New Brunswick, NJ and Evansville, IN; Schein Pharmaceuticals, Carmel, NY; SmithKline Beecham, King of Prussia, PA; and Barr Laboratories in Pomona, NY.

'I have been using Zymark products for more than 10 years and have been in the Zymark Productivity Club for three years', explained Muhammed Alburakeh, Manager, Quality Control-Robotics, Barr Laboratories. 'Automation has given us efficiency that we could never realize in our quality control operation. Zymark has worked with us to maximize our capabilities while asking for our input concerning product improvements'.

Using Zymark's MultiDose and MultiDose Plus Dissolution Workstations, TPW II Tablet Processing Workstations, Zymate Systems or BenchMate Sample Preparation Workstations, these award winners have found solutions to productivity and cost through automation. To see a complete list of winners and a pictorial review of the Award ceremony, visit the Productivity Award web site at www.zymark.com/MARKETIN/pdf/pdf.htm

Zymark Corporation, headquartered in Hopkinton, MA, is a worldwide leader in laboratory automation for pharmaceutical and biotechnology applications. Zymark serves the worldwide pharmaceutical and biotechnology industries, and hosts the annual ISLAR conference on laboratory automation. The company employs ~300 people in the USA, Canada, Europe and Japan, and has authorized sales and service distributors throughout the rest of the world.

*For further information contact: Lynda Thomas, Marketing Communications Specialist. Tel: + 1 508-497-6549; Fax: + 1 508-435-3439; e-mail: lynda.thomas@zymark.com*

